# Establishing a Student-Run Mobile Clinic in Appalachian Kentucky: A Community-Based Pilot Program to Address Health Inequities

**DOI:** 10.13023/jah.0604.08

**Published:** 2025-01-29

**Authors:** Bradley A. Firchow, John C. Cornelius

**Affiliations:** University of Kentucky College of Medicine; University of Kentucky College of Medicine

**Keywords:** Appalachia, free clinic, health equity, housing, mobile clinic, underserved

## Abstract

The establishment of a student-run mobile clinic in Morehead, Kentucky, led by students from the University of Kentucky College of Medicine’s (UKCOM) Rural Physician Leadership Program (RPLP), aims to address health inequities in rural Appalachian communities. This pilot program is designed to serve vulnerable populations, including the unhoused, by delivering free, accessible healthcare services directly to underserved populations. This program serves as a model for expanding access to healthcare in rural regions while promoting a collaborative, community-based approach to addressing social determinants of health.

Student-run clinics have emerged as a vital component of medical education and community health across the United States.^1^ They provide essential healthcare services to underserved populations while delivering valuable learning experiences for medical students.^2^,^3^ In rural regions like Appalachian Kentucky, where healthcare disparities are pronounced, these clinics play an even more critical role.^4^ This article describes the establishment of a student-run mobile clinic in Morehead, Kentucky, led by students from the University of Kentucky College of Medicine’s (UKCOM) Rural Physician Leadership Program (RPLP). The project aims to reduce health inequities among vulnerable populations, particularly the unhoused, by partnering on free and accessible healthcare services directly. It will serve as an initial access point for triage and referral to emergency and primary care. Research questions for this UKCOM Office of Community Advancement-funded pilot program include:

- How does providing mobile healthcare services influence patient satisfaction among vulnerable populations in a rural Appalachian setting? Baseline surveys will assess previous healthcare experiences, including frequency and quality of past access to care.^5^
- How do mobile healthcare services influence healthcare utilization among vulnerable populations in a rural Appalachian setting?^6^
- What are the strengths and challenges for implementing an interprofessional care model in a student-run mobile clinic serving rural, underserved communities?^7^
- Can the integration of evidence-based preventive and chronic disease management services improve health equity for vulnerable populations in rural areas? This will be measured by tracking blood pressure, HbA1c, and cholesterol metrics over time.^8^,^9^

## Context and Rationale

Rural Appalachia faces significant healthcare challenges, including high rates of chronic illness, limited access to healthcare providers, and overwhelming socioeconomic barriers.^10^ These issues are exacerbated for unhoused populations, who experience additional obstacles such as lack of transportation and distrust of the healthcare system.^11^ Student-run clinics have been shown to effectively address such barriers by offering community-based, patient-centered care tailored to the unique needs of vulnerable populations.^12^,^13^,^14^

However, few student-run clinics have addressed the unique challenges of rural populations, such as limited transportation and higher rates of chronic diseases, which are prevalent.^2^ The UKCOM – Morehead Mobile Clinic bridges this gap by partnering on care directly in patients’ living environments, eliminating transportation barriers that have historically restricted access to care in rural regions like Eastern Kentucky.^15^ By integrating evidence-based preventive services and addressing social determinants of health, the clinic seeks to enable health outcomes that were previously unattainable for these rural Appalachian residents.

## Design and Implementation

The clinic is designed to address both immediate healthcare needs and broader social determinants of health through a multi-faceted, evidence-based approach. Evidence-based practices like preventive screenings, chronic disease management, and mental health integration have been shown to significantly improve patient outcomes.^16^,^17^ The clinic provides point-of-care testing for HbA1c, cholesterol, and oxygen saturation, as well as diagnostic testing for flu, strep, and RSV, aligning with best practices for rural healthcare delivery. Mental health assessments, including PHQ-9 and GAD-7 screenings, capture the rising prevalence of depression and anxiety in underserved populations.^17^

The clinic’s interprofessional model fosters collaboration among medical, nursing, pharmacy, and physician assistant students, aligning with evidence-based approaches like the WHO’s Interprofessional Collaborative Practice Model to deliver holistic, coordinated care for underserved populations.^18^ While the current local programs do not include counseling, psychology, or social work students, the clinic aims to establish future partnerships to integrate these disciplines, as evidence demonstrates their critical contributions to addressing behavioral health and social determinants of health.^7^. Interprofessional education not only enhances the learning experience for students by exposing them to real-world teamwork dynamics but also directly benefits the community by improving care coordination and leveraging diverse expertise to meet patient needs. For example, pharmacy students can address medication adherence, while nursing students provide education on chronic disease self-management, creating a comprehensive care plan for patients.

Furthermore, local partnerships with the UK St. Claire Foundation, Gateway Homeless Coalition, and UK St. Claire Pharmacy provide essential resources such as free prescriptions, housing assistance, and food security programs. Research highlights the importance of community partnerships in student-run healthcare models, as these collaborations expand access to resources and facilitate comprehensive care.^19^ These partnerships not only align with evidence-based strategies for addressing social determinants of health but also strengthen the clinic’s capacity to deliver holistic, patient-centered care.

## Impact and Evaluation

The clinic’s impact will be assessed through a mixed-methods evaluation approach. A retrospective analysis of electronic medical records will be conducted beginning after one year of clinic operations, focusing on health outcomes, healthcare utilization, and patient satisfaction. The analysis will focus on a one-year pilot period, allowing sufficient time to evaluate the program’s effects while providing a structured timeframe for implementation and review.

Health outcomes will be measured compared to baseline and follow-up measurements of HbA1c, blood pressure, and cholesterol will be collected at 3– and 6-month intervals. Healthcare utilization will be measured from data on emergency department visits, referrals, and usage will be tracked to evaluate changes in utilization patterns within the UK hospital system. Patient satisfaction will be measured with surveys on perceived care quality, accessibility, and satisfaction, with a focus on comparing pre- and post-intervention experiences. Transportation will be assessed with geospatial analysis, which will measure average travel distances and time saved by accessing mobile clinic services compared to the UKSC Hospital and family medicine outpatient clinics. Monte Carlo simulations will estimate the economic and health impacts of preventive care interventions, including projected quality-adjusted life years (QALYs).^20^

This evidence-based approach ensures that the Morehead Mobile Clinic can adapt to meet the evolving needs of the community it serves. The simulation will incorporate baseline data gathered during initial assessments and follow-up measures taken over the study period, creating a dynamic model of cost-effectiveness and health improvement. By employing this evidence-based framework, the Morehead Mobile Clinic will be able to continuously refine its operations and adapt services to meet the evolving needs of the community.

## Challenges and Opportunities

Establishing a student-run mobile clinic in a rural, underserved area presents several challenges, including maintaining a consistent volunteer base, securing sustainable funding, and navigating the logistical complexities of mobile healthcare delivery.^2^ However, these challenges also present opportunities for innovation and collaboration. By engaging with community stakeholders and leveraging local resources, the Morehead Mobile Clinic aims to build a resilient and adaptable model of care that can be replicated in other rural communities facing similar healthcare disparities.

Volunteer recruitment will focus on engaging students, offering mentorship and leadership opportunities to retain participants. Retired healthcare professionals will also be invited to support clinic operations. The clinic will adopt a tiered structure, similar to Tulane University’s model, where senior students mentor juniors, fostering professional development and continuity of care.^3^

From a financial perspective, the Morehead Mobile Clinic adopts an adaptable model of care centered on sustainable funding through ongoing support from the UKCOM Office of Community Advancement and UK St. Claire Foundation, consistent volunteer engagement from health science students, and tailored service delivery. Drawing inspiration from successful programs including the Health Wagon in Virginia, the clinic will employ a mixed funding strategy combining grants and partnerships with local organizations like the St. Claire Foundation.

When designing logistics of the mobile clinic, predictable schedules and centralized locations improve accessibility, while partnerships with a co-located housing coalition will address broader social determinants of health.

## Conclusion

The Morehead Mobile Clinic addresses the healthcare needs of Appalachian Kentucky by providing free, accessible care tailored to the region, improving outcomes for vulnerable populations. By integrating holistic and evidence-based approaches, the clinic tackles both clinical symptoms and social determinants of health, including housing, transportation, and nutrition, while serving as a model for rural student-run clinics.

To ensure continuous improvement, the clinic employs retrospective EMR analysis and Monte Carlo simulations to refine services based on measurable outcomes like health improvements, quality-adjusted life years, and patient satisfaction. This evidence-based strategy, paired with ongoing research, community engagement, and interprofessional collaboration, fosters health equity and offers a sustainable model for addressing healthcare disparities in underserved rural areas, paving the way for a more equitable future in rural Appalachia.
